# Patient-reported outcome measures as determinants for the utilization of health care among outpatients with epilepsy: a prognostic cohort study

**DOI:** 10.1186/s41687-023-00641-4

**Published:** 2023-10-20

**Authors:** Stine Primdahl Rasmussen, Liv Marit Valen Schougaard, Niels Henrik Hjøllund, David Høyrup Christiansen

**Affiliations:** 1 Department of Occupational Medicine, Danish Ramazzini Centre, Gødstrup Hospital, Hospitalsparken 15, Herning, 7400 Denmark; 2 AmbuFlex - Center for Patient-reported Outcomes, Central Denmark Region, Gødstrup Hospital, Møllegade 16, Herning, 7400 Denmark; 3 Department of Clinical Medicine, Health, Aarhus University, Palle Juul-Jensens Blvd. 82, Aarhus, 8200 Denmark; 4 Elective Surgery Centre, Silkeborg Regional Hospital, Falkevej 1A, Silkeborg, 8600 Denmark; 5 Centre for Research in Health and Nursing, Research, Regional Hospital Central Jutland, Heibergs Allé 2K, Viborg, 8800 Denmark

**Keywords:** Patient-reported Outcomes Measures, Use of Health Care Services, Epilepsy, Outpatient care, Health literacy

## Abstract

**Background:**

Patient-reported outcome (PRO) measures can inform clinical decision making and planning of treatment in the health care system. The aim of this study was to examine whether patient-reported health domains influence the use of health care services in outpatients with epilepsy.

**Methods:**

This was a prognostic cohort study of 2,426 epilepsy outpatients referred to PRO-based follow-up at the Department of Neurology, Aarhus University Hospital, Denmark. Patients filled out a questionnaire covering health literacy areas, self-efficacy, well-being and general health. The main outcome was a record of contact to the epilepsy outpatient clinic, inpatient ward and/or emergency room within 1 year, retrieved from health register data. Associations were analysed by multivariable binomial logistic regression.

**Results:**

A total of 2,017 patients responded to the questionnaire and 1,961 were included in the final analyses. An outpatient contact was more likely among patients with very low health literacy (‘social support’): odds ratio (OR) 1.5 (95% CI: 1.1–2.1), very low and low self-efficacy: OR 1.7 (95% CI: 1.2–2.3) and OR 1.4 (95% CI: 1.0–1.8), low and medium well-being: OR 2.2 (95% CI: 1.6–3.0) and OR 1.4 (95% CI: 1.1–1.9), and patients rating their general health as fair: OR 2.8 (95% CI: 1.7–4.6). Inpatient contact and emergency room contact were associated with the health domains of self-efficacy and general health.

**Conclusions:**

PRO questionnaire data indicated that patients with low health literacy (“social support”), well-being, self-efficacy and self-rated general health had an increased use of health care services at 1 year.These results suggest that PRO measures may provide useful information in relation to the possibility of proactive efforts and prevention of disease-related issues and to help identify efficiency options regarding resource utilization.

**Supplementary Information:**

The online version contains supplementary material available at 10.1186/s41687-023-00641-4.

## Abstract (plain language summary)

The use of patient reported outcomes (PRO) measures to monitor and plan treatment in health care has become increasingly common in recent years. In this study, we examined whether PRO measures of patients self-perceived levels of health knowledge, confidence in managing their health, overall sense of well-being, and general perception of their health could predict the need for contact in an epilepsy outpatient clinic during a 12 months’ period. An outpatient contact was more likely among patients with lower levels of health literacy, well-being, self-efficacy and self-rated general health. The study indicates that PRO measures may hold useful information to inform clinical decision making and planning of care among outpatients with epilepsy.

**Supplementary Information:**

The online version contains supplementary material available at 10.1186/s41687-023-00641-4.

## Background

Epilepsy is a chronic neurological disease characterized by recurrent seizures that affects approximately 0.5–1% of the world’s population and is seen in all age groups [1]. Epilepsy has a major influence on individuals and an impact on society [1, 2]. Epilepsy and the frequency of seizures are associated with increased risk of depression [3] and reduced quality of life [4]. In Denmark, people with epilepsy receive more social services, have a lower employment rate and significantly higher mortality than people without epilepsy [2]. Seizure frequency is strongly related to the use of health care resources [5], and people with epilepsy are more frequently in contact with health care service providers than many other patient groups seeking health care [6]. A Danish study from 2016 found that 56.4% of people with epilepsy used outpatient services as compared to 29.9% in a matched control group [2]. Other studies have shown that people with epilepsy are more frequently in contact with hospitals and emergency rooms than healthy control subjects and even people suffering from other chronic diseases [7]. Moreover people with epilepsy are more than twice as likely to contact the hospital and general practice compared to people without epilepsy [8].

The use of patient-reported outcomes (PROs) in clinical practice has become increasingly common in recent years [9]. PROs are defined as “… any report of the status of a patient’s health condition that comes directly from the patient, without interpretation of the patient’s response by a clinician or anyone else.” [10]. Since PROs are subjective, they can be used to provide information about non-observable constructs such as health literacy, self-efficacy, general health and well-being.

The concept of health literacy has been defined as: a person’s ability to understand and use information to make decisions about their health” [11]. However, several different health literacy measurement methods have been used and divergent results regarding whether health literacy is associated with the use of health care have been reported [12–19]. In two recent Danish studies, high health literacy measured by using the Health Literacy Questionnaire was shown to have a significant positive association with healthy behaviour among patients with diabetes and cardiovascular diseases [20, 21].

In 2015, an American study demonstrated that low self-rated general health on the day of hospital discharge was predictive of re-utilization within 14 days among general medical and intensive care patients [22]. These findings suggest a link between health domains measurable by PROs and health care utilization, which may reflect that patients have different needs for contact with the health care system. Whether PRO measures can inform on patients’ need for contact has to our knowledge not been studied among epilepsy outpatients.

The aim of this study was to examine whether PRO-assessed health literacy, self-efficacy, well-being and general health status were independently associated with seeking health care in outpatients with epilepsy. We hypothesized that patients with low levels in these domains would be more likely to seek outpatient health care services.

## Methods

### Design

This study was conducted as a prognostic cohort study with one-year register based follow up data [23–25].

### The AmbuFlex system

This study was conducted within the context of the AmbuFlex system. AmbuFlex is a generic clinical application developed in Central Denmark Region that supports PRO-based outpatient follow-up. The aim of AmbuFlex is to support clinical decision-making, improve quality of care and achieve greater flexibility and ability to prioritize resources to outpatients with actual need or wish for clinical attention [9, 26–28]. AmbuFlex is currently applied to more than 50 diagnostic groups.

AmbuFlex/Epilepsy has been in operation since 2012, and by April 2021, 2,983 patients had been referred to AmbuFlex/Epilepsy in three neurological outpatient clinics in Central Denmark Region. In PRO-based follow-up, patients report disease-specific symptoms and health status from home instead during traditional outpatient follow-up with fixed appointments Patients are referred to AmbuFlex as a part of their treatment, based on other formal inclusion criteria than the clinician’s subjective clinical judgement and the patient’s own preferences for attending PRO-based follow-up and their consent to participate [29]. There are no pre-scheduled follow-ups, although outpatients can always phone the clinic if necessary. Patients with cognitive limitations can be referred to similar questionnaires developed for proxy use, where relatives or health professionals assist with the replies.

**Fig. 1:**
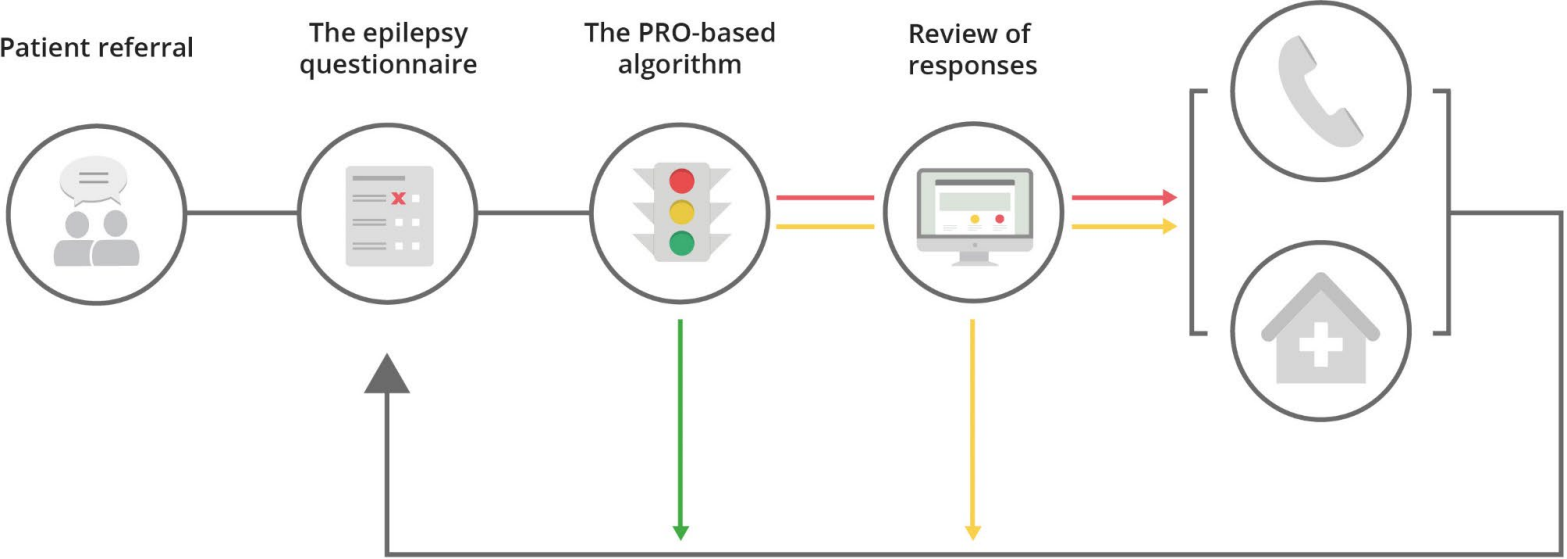
Overview of the principles of the AmbuFlex System

Each referred patient is prompted to fill out the questionnaires every 3rd, 6th or 12th month, and responses are handled by a PRO-based automatic decision algorithm [30] (Fig. 1). Based on pre-defined thresholds, patients are divided into two groups: whether they need or wish clinical attention or not. Each response category for each question is assigned a colour code: red, yellow or green. A green code for all responses means no need or wish for contact. Clinicians assess yellow-coded responses with additional information from the medical record system, and evaluate whether the patient is to be contacted. For example, a yellow code can be the reported presence of one or more symptoms. The patient must be contacted in the case of a red-coded response, for example, reported aggravation of seizures or planning of pregnancy. One of the questions is whether the patient feels the need for contact, and if they do, this overrules any automated decision. A graphical overview of the patients’ responses is integrated into the electronic health record to support the clinical decision [26].

### Settings and participants

The source population for the analyses was outpatients with epilepsy referred to PRO-based follow-up at the Department of Neurology, Aarhus University Hospital, Denmark. From January 2016 to January 2017, the patients were sent an additional research questionnaire combined with a scheduled epilepsy questionnaire. The additional questionnaire contained information concerning personal and health-related aspects including health literacy, well-being, self-efficacy and general health. The index date of the observation period was the return date of the additional questionnaire, which was filled out electronically or by paper. A 7-day shipment period was added to the index date of paper replies. Participants were excluded in the case of death or migration within the follow-up time. Subsequently, the associations between the health domains and contact to health care services were examined.

### Ethics approval and consent to participate

The Danish Data Protection Agency approved the PhD study (reference number 1-16-02-691-14) from which data were used [9]. According to Danish law, approval by the ethics committee and written informed consent was not required [31]. The eligible patients were provided with information about the study and its purpose, including that participation was voluntary and how they could opt out if so wished.

### Outcome

Measured 1 year from the index date of the observation period, we extracted all records of contacts under primary and a secondary ICD-10 diagnosis of epilepsy from the outpatient clinic and the inpatient ward at the Department of Neurology and the emergency room at Aarhus University Hospital. Data were obtained from the Business Intelligence Register in Central Denmark Region, which contains information about current or previous patients in Central Denmark Region, defined by individual unique personal numbers. Contact with the epilepsy outpatient clinic included face-to-face or telephone consultations with physicians or nurses, and administrative contact by web/e-mails or by letter. Contact with the inpatient ward included hospitalizations, and emergency room contact included epilepsy-related emergency visits. The outcome was the patients need for contact defined as at least one contact (i.e. ≥ 1 contact versus no contact) within the follow period.

### Potential determinants

*Health Literacy*. The Health Literacy Questionnaire (HLQ) is a 44-item questionnaire divided into nine conceptual domain scales of health literacy. The development of HLQ was validity-driven and consisted in-depth grounded consultations, psychometric analysis and cognitive interviews [32]. It has been translated into Danish according to international standards [33]. The subscales can be used independently and the three following subscales of the HLQ were considered relevant and included in the present study: “Social Support for health”, “Ability to actively engage with health care providers” and “Understanding health information well enough to know what to do”. Each area is divided into subscales. The scale “Social Support for health” (HLQ4) contains five items with four response options (strongly disagree, disagree, agree, strongly agree), which are averaged to a 1 to 4 (High support ) score. The scales covering the domains of “Ability to actively engage with health care providers” (HLQ6) and “Understanding health information well enough to know what to do” (HLQ9) each include five items with five response options (cannot do, very difficult, quite difficult, easy, very easy) averaged to a 1 to 5 (best) score.

*Well-being*. The WHO-Five Well-Being Index (WHO-5) is a five-item questionnaire measuring subjective psychological well-being and has psychometric properties that have been assessed in several chronic diseases [34–37]. Every item has six-scaled response options ranging from “At no time” to “All of the time”, each scored from 0 to 5. The sum of the response options is multiplied by 4, giving a total score range of 0–100, with higher scores representing better self-assessed well-being and score below 50 indicating risk of depression [35].

*Self-Efficacy*. The General Self-Efficacy scale (GSE) is a 10-item psychometric questionnaire designed to assess one’s belief in own competence to cope with difficult demands, and the psychometric properties have been assessed in different countries and populations [38, 39]. Each item has a score from 1 to 4 (not at all true, hardly true, moderately true, exactly true). The GSE has a total score range of 10–40. High score means better self-assessed self-efficacy.

*General health*. The Short Form Health Survey (SF-36) measures health-related quality of life in a 36-item questionnaire [40]. Only the first item was included in this study: “In general, would you say your health is” and had five response options (excellent, very good, good, fair, poor).

### Potential confounders

To control for the potential influence of other factors, we include five additional variables: age, gender, educational level, years with epilepsy diagnosis and seizure frequency obtained from either the AmbuFlex system or by questionnaire [6, 8, 12–15].

The variable, educational level, was divided into three categories: low (no vocational education or one or more shorter courses), middle (1–4 years of study or vocational education) and high (more than 4 years of study). Years with diagnosis of epilepsy was a dichotomous variable defined </≥ 2 year, and seizure frequency was defined </≥ 1 seizure during the last 12 months.

### Statistical analysis

Patient characteristics were presented with descriptive variables by number and percentage for categorical variables, whereas continuous variables were presented by mean and standard deviation (SD) or median and inter quartile range (IQR) depending on their distribution. Wilcoxon rank sum test and chi-square test were used to assess potential age and gender differences between respondents and non-respondents. Next, the associations between each potential determinant and outcome variable (≥ 1 contact versus no contact) were analysed by binomial logistic regression. Age, sex, educational level, years with epilepsy diagnosis and seizure frequency during the last year were added as potential confounders in adjusted analyses. Assumptions for numbers of variables needed to avoid over-fitting the model were based on the rule-of-thumb of at least 10 cases per variable [41]. Estimates were reported as odds ratios (ORs) with 95% confidence intervals (CIs). Prior to the analyses, assumptions for binomial regression were controlled for. The health domains were categorized if the log-odds were not a linear function of the health domains. All HLQ variables and the GSE were categorized at the 0–24th (very low), 25–49th (low), 50–74th (medium) and 75–100th (high) percentile. WHO-5 was categorized as scores of 0–49 (low), 50–69 (medium) and 70–100 (high).

Supplemental analyses were performed by excluding 339 patients, who were allocated to patient-initiated follow-up as part of a randomized controlled study conducted within the AmbuFlex system [9, 42]. Participants in that group did not receive pre-scheduled questionnaire during the follow-up period and therefore may differ with respect to health care contacts. The statistical analyses were conducted with STATA version 16.1 (StataCorp., College Station, TX, USA).

## Results

### Characteristics of participants

**Fig. 2:**
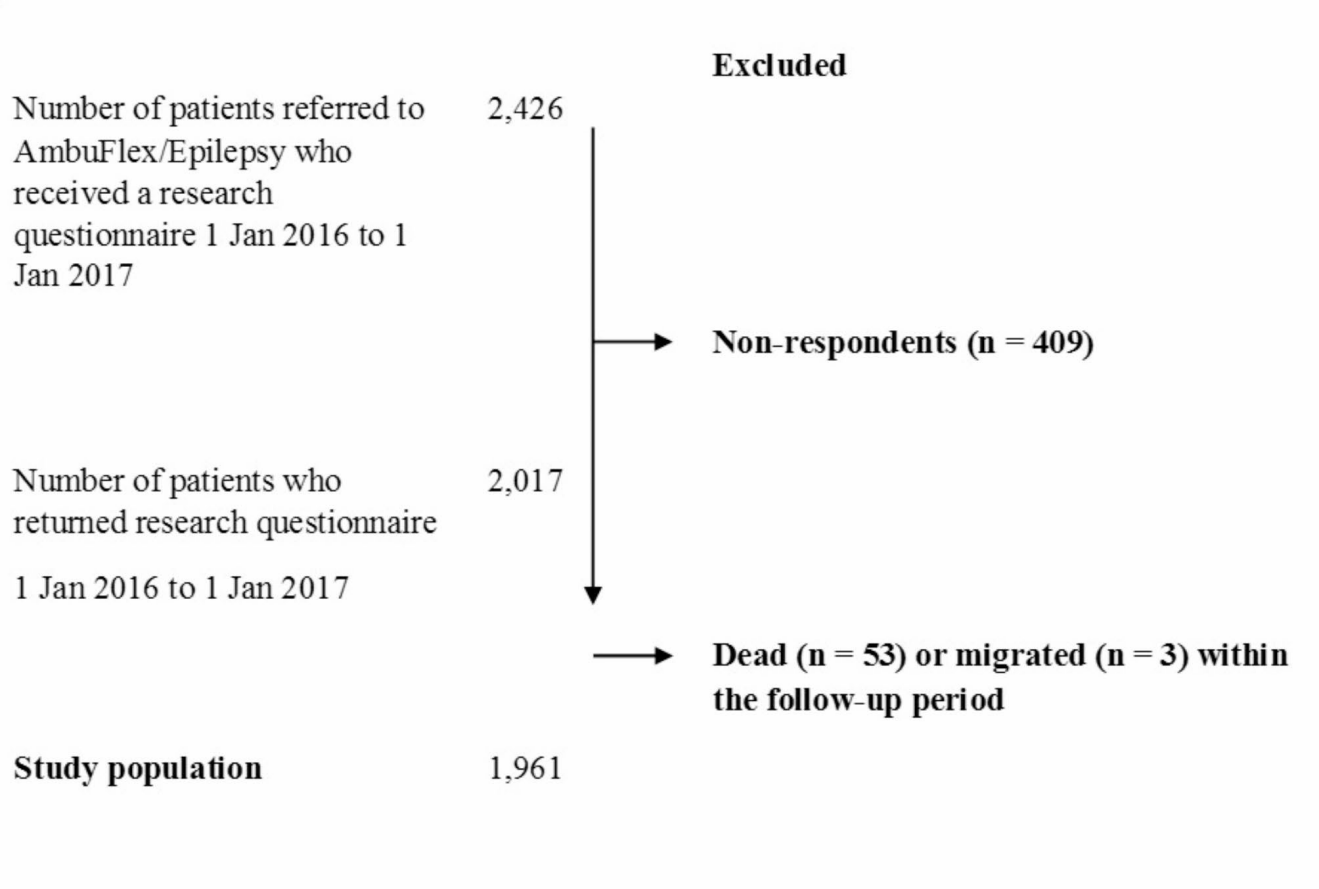
Flowchart of outpatients included from the Department of Neurology, Aarhus University Hospital, Aarhus, Denmark

A total of 2,426 patients were sent the additional questionnaire in the period from January 2016 to January 2017, and 2,017 (83.1%) patients responded (Fig. 2). After excluding patients due to death (n = 53) or migration (n = 3), the study population comprised 1,961 patients. There was no significant difference in gender between respondents and non-respondents (p = 0.13). Non-respondents were significantly younger than respondents (p = 0.03), mean age 47.6 (SD 19.1) and 49.7 (SD 18.6), respectively. The characteristics of the included patients are presented in Table 1. In total 1,487 patients had the epilepsy diagnose for 2 or more years and 520 had had one or more seizures during the last year. More than half of the study population had at least one epilepsy outpatient clinic contact during the year, whereas only 80 (4.1%) patients had an inpatient contact, and 155 (7.9%) had an emergency room contact.

**Table 1:** Baseline characteristics of outpatients with epilepsy from the Department of Neurology, Aarhus University Hospital, Aarhus, Denmark (N = 1961)

Gender, *n (%)*		
Male Female	978 983	(49.9) (50.1)
Age, years, *mean (SD)*	49.9	(18.4)
Social support for health (HLQ, 4), *Median (IQR)* Missing, *n (%)*	3.4 142	(0.8) (7.2)
Ability to actively engage with health care providers (HLQ, 6), *Median (IQR)* Missing, *n (%)*	4.0 145	(1.0) (7.4)
Understanding health information (HLQ, 9), *Median (IQR)* Missing, *n (%)*	4.0 146	(1.2) (7.4)
General self-efficacy (GSES), *Median (IQR)* Missing, *n (%)*	30.0 162	(8.0) (8.3)
Well-being (WHO-5), *Median (IQR)* Missing, *n (%)*	76.0 146	(20.0) (7.4)
General health (one item from SF36), *n (%)*		
**Excellent** **Very good** Good Fair Poor Missing, *n (%)*	185 663 778 241 61 33	(9.4) (33.8) (39.7) (12.3) (3.1) (1.7)
Years with diagnose of epilepsy, *n (%)*		
≥ 2 years < 2 years Missing, *n (%)*	1,487 109 365	(75.8) (5.6) (18.6)
Educational level, *n (%)*		
High Middle Low Missing, *n (%)*	169 1,136 509 147	(8.6) (57.9) (26.0) (7.5)
Seizure frequency, *n (%)*		
**≥ 1** < 1 Missing, *n (%)*	520 1316 125	(26.5) (67.1) (6.3)
Response type, *n (%)*		
Paper Web	693 1,268	(35.3) (64.7)
Departmental contact, *n (%)*		
Outpatient clinic, Neurological Department Inpatient ward, Neurological Department Emergency Department	1,083 80 155	(55.2) (4.1) (7.9)

SD = Standard deviation, IQR = Inter quartile range

### Missing values

At least one answer was missing for 142 (7.2%) patients in the social support for health scale (HLQ4), 146 (7.4%) patients in the WHO-5, Ability to actively engage with health care providers scale (HLQ6) and Understanding health information scale (HLQ9), 162 (8.3%) patients in the GSE and 33 (1.7%) patients in the general health question (SF-36). A total of 365 (18.6%) patients did not disclose the year of diagnosis, 147 (7.5%) patients did not answer the education question, and 125 (6.3%) patients did not answer the seizure frequency question.

### Associations between health domains and outpatient contact

**Fig. 3:**
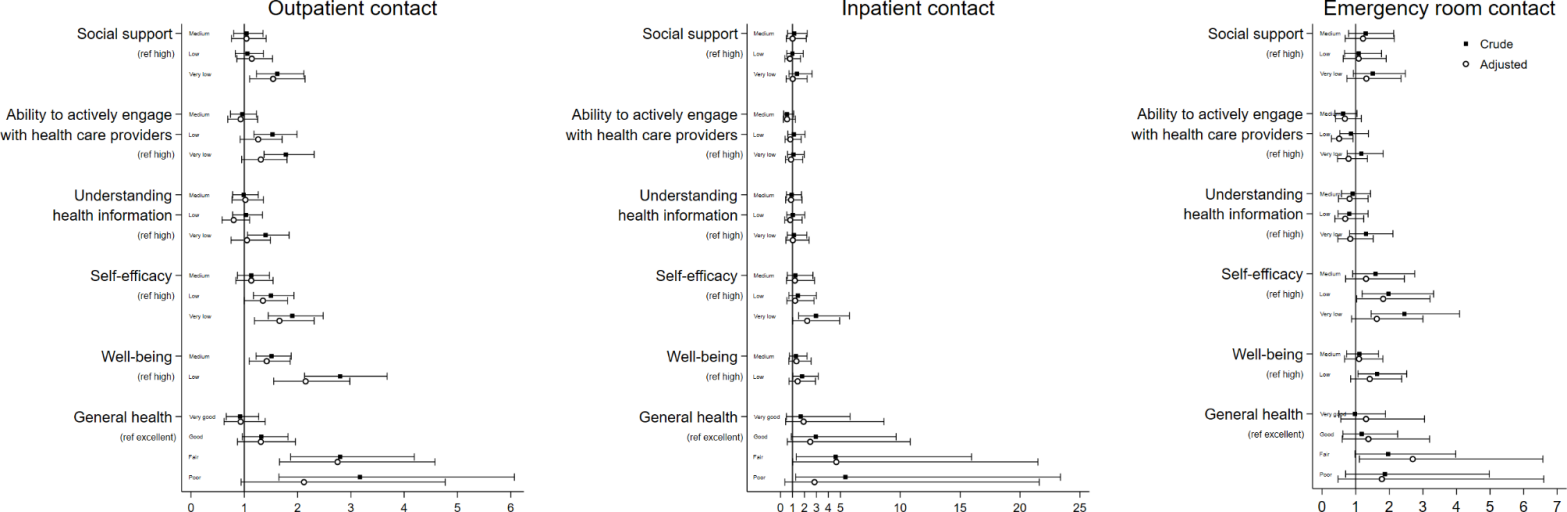
Associations between health care contacts and PRO measures in outpatients with epilepsy

Patients with very low HLQ4 scores (social support for health) were more likely to have an outpatient contact than patients who had high HLQ4 scores (OR 1.54, 95% CI: 1.10–2.14) (Table 2; Fig. 3). Similar findings were observed with respect to GSE (self-efficacy) scores (Table 2). For well-being scores (WHO-5) having low and medium scores was associated with an outpatient contact: adjusted OR of 2.15 (95% CI: 1.55–2.98) and 1.42 (95% CI: 1.09–1.86). Patients rating their health condition as fair was also more likely to have and outpatient contact, when compared to patients with excellent health: adjusted OR of 2.75 (95% CI: 1.66–4.58). The ability to understand health information well enough to know what to do (HLQ9) and the ability to actively engage with health care providers (HLQ6) was not statistically significantly associated with an outpatient contact in the adjusted analysis.

**Table 2:** Associations between the need for outpatient contact and PRO measures in outpatients with epilepsy

Potential Determinants	Cases		OR	95% CI	Adjusted OR ^a^	95% CI
	n	%				
Social support for health (HLQ4 scores)		(n = 1,819)^b^		(n = 1,441)^b^		
High (score 3.8-4.0) Medium (score 3.4–3.7) Low (score 3.0-3.3) Very low (score 1-2.9)	271 217 280 233	52 54 54 64	1 1.04 1.06 1.62	- 0.80 – 1.35 0.83 – 1.36 1.23 – 2.12	1 1.04 1.14 1.54	- 0.76 – 1.41 0.86 – 1.53 1.10 – 2.14
Ability to actively engage with health care providers (HLQ6 scores)		(n = 1,816)^b^		(n = 1,437)^b^		
High (score 4.4-5.0) Medium (score 4.0-4.3) Low (score 3.4–3.9) Very low (score 1.0-3.3)	269 220 245 267	50 49 61 64	1 0.96 1.53 1.78	- 0.74 – 1.23 1.18 – 1.99 1.37 – 2.31	1 0.93 1.26 1.31	- 0.69 – 1.25 0.92 – 1.71 0.95 – 1.80
Understanding health information (HLQ9 scores)		(n = 1,815)^b^		(n = 1,444)^b^		
High (score 4.6-5.0) Medium (score 4.0-4.5) Low (score 3.4–3.9) Very low (score 1.0-3.3)	252 310 210 233	54 54 55 62	1 0.99 1.03 1.40	- 0.78 – 1.26 0.78 – 1.34 1.06 – 1.84	1 1.02 0.80 1.05	- 0.77 – 1.36 0.58 – 1.10 0.75 – 1.49
Self-efficacy (GSE scores)		(n = 1,799)^b^		(n = 1,431)^b^		
High (score 33–40) Medium (score 30–32) Low (score 25–29) Very low (score 10–24)	240 207 281 261	48 52 59 64	1 1.13 1.50 1.90	- 0.87 – 1.47 1.17 – 1.93 1.45 – 2.48	1 1.13 1.35 1.66	- 0.84 – 1.54 1.00 – 1.81 1.19 – 2.31
Well-being (WHO5 scores)		(n = 1,815)^b^		(n = 1,389)^b^		
High (score 70–100) Medium (score 50–69) Low (score 0–49)	491 285 233	49 59 73	1 1.51 2.80	- 1.22 – 1.88 2.13 – 3.68	1 1.42 2.15	- 1.09 – 1.86 1.55 – 2.98
General health (SF-36 first item categories)		(n = 1,928)^b^		(n = 1,470)^b^		
Excellent Very good Good Fair Poor	91 312 436 176 46	50 48 56 73 76	1 0.92 1.32 2.80 3.17	- 0.66 – 1.27 0.96 – 1.82 1.87 – 4.19 1.65 – 6.07	1 0.93 1.31 2.75 2.12	- 0.62 – 1.39 0.87 – 1.96 1.66 – 4.58 0.94 – 4.77

% = Percentage of patients who had outpatient contact within potential determinant category, CI = Confidence Interval

^a^ Adjusted for age, gender, educational level, years with diagnose and seizure frequency

^b^ Total numbers included in models as numbers vary due to missing values

### Associations between health domains, inpatient contact and emergency room contact

The associations between health domains and an inpatient and emergency room contact resented in Fig. 3; Tables 3 and 4, respectively. As measured by the GSE, patients with very low self-efficacy had statistically higher odds of inpatient contact (adjusted OR: 2.23, 95% CI: 1.01–4.94) when compared to patients with high self-efficacy (Table 3). Compared to patients with excellent health, patients with fair health were more likely to have an inpatient contact (adjusted OR: 4.66, 95% CI: 1.01–21.49). None of the three health literacy areas or well-being had a statistically significant association with an inpatient contact in the adjusted analyses. Emergency room contact were more often seen among patients with low self-efficacy scores (GSE), than patients with high self-efficacy scores: adjusted OR 1.82 (95% CI: 1.03–3.21) (Table 4). Compared to patients with self-rated excellent general health, those with self-rated fair general health also had more frequent emergency room contact: adjusted OR 2.70 (95% CI: 1.11–6.57). None of the three health literacy areas or well-being had a statistically significant association with having an emergency room contact in the adjusted analyses.

**Table 3:** Associations between the need for inpatient contact and PRO measures in outpatients with epilepsy

Potential Determinants	Cases		OR	95% CI	Adjusted OR ^a^	95% CI
	n	%				
Social support for health (HLQ4 scores)		(n = 1,819)^b^		(n = 1,441)^b^		
High (score 3.8-4.0) Medium (score 3.4–3.7) Low (score 3.0-3.3) Very low (score 1.0-2.9)	19 17 19 18	4 5 4 5	1 1.15 1.00 1.37	- 0.59–2.23 0.52–1.90 0.71–2.64	1 1.01 0.79 1.02	- 0.48–2.15 0.37–1.67 0.47–2.22
Ability to actively engage with health care providers (HLQ6 scores)		(n = 1,816)^b^		(n = 1,437)^b^		
High (score 4.4-5.0) Medium (score 4.0-4.3) Low (score 3.4–3.9) Very low (score 1.0-3.3)	24 11 20 20	5 3 5 5	1 0.54 1.11 1.08	- 0.26–1.11 0.61–2.05 0.59–1.98	1 0.55 0.82 0.88	- 0.24–1.24 0.38–1.73 0.42–1.86
Understanding health information (HLQ9 scores)		(n = 1,815)^b^		(n = 1,444)^b^		
High (score 4.6-5.0) Medium (score 4.0-4.5) Low (score 3.4–3.9) Very low (score 1.0-3.3)	19 22 16 17	4 4 5 5	1 0.93 1.02 1.12	- 0.50–1.75 0.52–2.02 0.57–2.19	1 0.88 0.81 1.03	- 0.43–1.79 0.36–1.80 0.45–2.38
Self-efficacy (GSE scores)		(n = 1,799)^b^		(n = 1,431)^b^		
High (score 33–40) Medium (score 30–32) Low (score 25–29) Very low (score 10–24)	13 13 18 30	3 4 4 8	1 1.24 1.45 2.96	- 0.57–2.71 0.70–2.99 1.52–5.75	1 1.20 1.22 2.23	- 0.50–2.85 0.53–2.80 1.01–4.94
Well-being (WHO5 scores)		(n = 1,815)^b^		(n = 1,389)^b^		
High (score 70–100) Medium (score 50–69) Low (score 0–49)	36 22 20	4 5 7	1 1.29 1.80	- 0.75–2.21 1.03–3.15	1 1.33 1.43	- 0.69–2.55 0.71–2.92
General health (SF-36 first item categories)		(n = 1,928)^b^		(n = 1,470)^b^		
Excellent Very good Good Fair Poor	3 18 36 17 5	2 3 5 8 9	1 1.69 2.94 4.60 5.42	- 0.49–5.81 0.90–9.66 1.33–15.95 1.26–23.38	1 1.93 2.48 4.66 2.84	- 0.43–8.64 0.57–10.83 1.01–21.49 0.37–21.60

% = Percentage of patients who had inpatient contact within potential determinant category, CI = Confidence interval

^a^ Adjusted for age, gender, educational level, years with diagnose and seizure frequency

^b^ Total numbers included in models as numbers vary due to missing values

**Table 4:** Associations between the need for emergency room contact and PRO measures in outpatients with epilepsy

Potential Determinants	Cases		OR	95% CI	Adjusted OR ^a^	95% CI
	n	%				
Social support for health (HLQ4 scores)		(n = 1,819)^b^		(n = 1,441)^b^		
High (score 3.8-4.0) Medium (score 3.4–3.7) Low (score 3.0-3.3) Very low (score 1.0-2.9)	34 34 37 35	10 8 9 7	1 1.30 1.09 1.51	- 0.79–2.13 0.67–1.77 0.93–2.48	1 1.22 1.09 1.32	- 0.69–2.15 0.63–1.91 0.74–2.35
Ability to actively engage with health care providers (HLQ6 scores)		(n = 1,816)^b^		(n = 1,437)^b^		
High (score 4.4-5.0) Medium (score 4.0-4.3) Low (score 3.4–3.9) Very low (score 1.0-3.3)	46 25 30 41	9 6 8 10	1 0.63 0.86 1.17	- 0.38–1.04 0.53–1.38 0.75–1.82	1 0.68 0.51 0.79	- 0.40–1.17 0.28–0.92 0.46–1.35
Understanding health information (HLQ9 scores)		(n = 1,815)^b^		(n = 1,444)^b^		
High (score 4.6-5.0) Medium (score 4.0-4.5) Low (score 3.4–3.9) Very low (score 1.0-3.3)	37 42 25 38	8 8 7 11	1 0.91 0.81 1.31	- 0.58–1.44 0.48–1.37 0.82–2.11	1 0.82 0.69 0.84	- 0.49–1.37 0.38–1.24 0.47–1.52
Self-efficacy (GSE scores)		(n = 1,799)^b^		(n = 1,431)^b^		
High (score 33–40) Medium (score 30–32) Low (score 25–29) Very low (score 10–24)	24 30 44 45	3 8 10 11	1 1.59 1.98 2.45	- 0.91–2.76 1.19–3.32 1.46–4.09	1 1.31 1.82 1.63	- 0.70–2.45 1.03–3.21 0.88–3.00
Well-being (WHO5 scores)		(n = 1,815)^b^		(n = 1,389)^b^		
High (score 70–100) Medium (score 50–69) Low (score 0–49)	70 37 35	7 8 11	1 1.11 1.64	- 0.73–1.68 1.07–2.52	1 1.10 1.42	- 0.67–1.81 0.85–2.37
General health (SF-36 first item categories)		(n = 1,928)^b^		(n = 1,470)^b^		
Excellent Very good Good Fair Poor	12 42 59 29 7	7 7 8 12 12	1 0.98 1.18 1.97 1.87	- 0.50–1.89 0.62–2.25 0.98–3.98 0.70–4.98	1 1.31 1.38 2.70 1.78	- 0.56–3.05 0.60–3.20 1.11–6.57 0.48–6.60

% = Percentage of patients who had emergency room contact within potential determinant category, CI = Confidence interval

^a^ Adjusted for age, gender, educational level, years with diagnose and seizure frequency

^b^ Total numbers included in models as numbers vary due to missing values

### Supplemental analyses

The results of the analyses excluding participants with no pre-schedule PRO follow-up in the randomized controlled study are presented in supporting information S1 Appendix Tables 5, 6 and 7. The results of these analyses differentiated only slightly from our main analysis with respect to outpatient contacts. However, associations increased slightly for low ability to actively engage with health care providers and patients with poor general health (see S1 Appendix, Table 5). With respect inpatient and emergency room contacts the supplemental analyses differed only slightly with respect to point estimates, however precision of this analysis decreased and none of the associations reached statistical significance (S1 Appendix, Tables 6 and 7).

## Discussion

The use of health care outpatient services was more frequent among patients with low levels of health literacy (social support for health), well-being, self-efficacy and self-rated general health. These findings are in line with our specified hypothesis. Contrary to this study’s hypothesis, the observed associations between an outpatient contact and the two health literacy domains “ability to actively engage with health care providers” and “understanding health information well enough to know what to do” failed to reach statistical significance. Only a few associations with an inpatient and emergency room contact and health domains were statistically significant. These findings may indicate that PRO measures provides useful additional information in terms of proactive efforts and planning health care services in patients with epilepsy. In comparison to our findings the study by Howard et al. found similar associations between levels of health literacy and the outpatient contact [13] and a more recent study found that epilepsy outpatients with low levels of health literacy, self-efficacy, well-being, or general health were less likely to be referred remote care [29]. In contrast to our findings, that study reported a positive association for all three health literacy domains (HLQ 4, 6 and 9), whereas we only observed a positive association between lower levels of social support for health (HLQ4) and outpatient contacts. In line with our study Vandenbosch et al. reported a significant association between use of a 1-day clinic and lower levels of social support for health [14]. Howard et al. also observed a significant positive association between inadequate health literacy and being an inpatient, and several other studies have found a significant association between inadequate health literacy and emergency room contact, which is in contrast to our findings [12, 13, 15]. These differences between studies may be explained by different designs, patient populations and sizes of the study population as well as the fact that this study examined specific subscales of the Health Literacy Questionnaire and health literacy was measured with diverse tools in the aforementioned studies. In addition, we chose to categorize the HQL scales by quartiles, whereas most previous studies have analysed the scale continuously [21, 43, 44] or dichotomized the scale to identify persons who disagreed versus agreed to having social support and found it difficult versus easy to actively engage with health care providers and understand health information [20, 45].

In accordance with our findings, Chamberlain et al. examined the first question of self-rated general health in the 12-item Short Form Health Survey as a predictor for use of health care services among patients with heart failure [46], but no association between self-rated general health and outpatient visits was found. However, patients with poor and fair self-rated general health had a significantly increased risk of hospitalization and contact to the emergency department compared to patients with “good–excellent” general health [46]. Those findings partly reflect the findings in our study. The different findings regarding outpatient contact may be due to poor and fair self-rated general health in patients with heart failure because this disease could be considered more life threatening and therefore the patients would have more hospitalizations and contact to the emergency room. That would also explain the stronger associations with hospitalizations and contact to the emergency room for poor and fair self-rated general health compared to excellent self-rated general health among patients with heart failure compared to patients with epilepsy.

### Strengths and limitations

The study population is most likely a representative sample of the population of AmbuFlex/Epilepsy at Aarhus University Hospital because almost the entire population, except those with proxy questionnaires, were included. With a relative high participation rate (83.1%), we believe the risk of a systematic selection into to the study to be limited. Although, non-responders were significantly younger than the responders, it seems unlikely that young non-respondents differed from young responders with respect to our outcome variable – i.e. future contacts to health care services.

A strength of the study is the prospective design, and almost complete data were available through registers within the follow-up period, with only 3% of the patients lost to follow-up due to death or migration. The disadvantage of collecting information on hospital contact via a register is that registers are collected for administrative purposes and not for research, which may result in a lower quality of data. However, missing registration of contact in relation to epilepsy probably has personal consequences for the patients involved and must therefore be limited. Our categorization of health outcomes resulted in few cases of patients with a health care contact. This of course increased the uncertainty of the estimates especially for the results of inpatient contact and emergency room contact, where broad confidence intervals question the statistical strength – even though estimates were significant. These associations no longer reached statistical significance, when excluding participants who did not receive pre-schedule PRO in our supplemental analyses. However, overall our supplemental analyses did not differ substantially from the main analyses and we therefore believe this to be a result of a low number of cases in these outcomes rather than a systematic effect of patient-initiated follow-up [42].

Similar categorization of potential confounders and missing items regarding educational level, years with diagnosis of epilepsy and seizure frequency may have led to some residual confounding. Furthermore, there may be recall bias regarding years with a diagnosis of epilepsy because it was self-reported. There would probably have been fewer missing data regarding years with a diagnosis of epilepsy if the information had been collected from hospital records, although this method requires more resources and time. Other factors such as living alone and number of chronic conditions may also have been relevant in the analyses, as they have been associated with different levels of sub-scale 4 in the HLQ. Beauchamp et al. found that people living alone and with ≥ 4 chronic conditions reported having less social support for health [45]. However, inclusion of further variables would have required a larger sample size to avoid an overfitted regression model and erratic estimates. Furthermore, a high number of statistical tests were performed to explore the associations between a range of health domains and outpatient contacts, which to some extend could have increased the chance of false positive findings (i.e. type 1 error). Finally, the explanatory nature of the study should be noted. The aim of current study was only to provide evidence supportive of the independent effect of the different health domains, while controlling for known confounders [25]. Thus, the individual contribution of these variables for the overall prediction of health care outpatient services should be further investigated. The results of this study can be generalized to almost all outpatients in AmbuFlex/Epilepsy at the Department of Neurology, Aarhus University Hospital. However, further generalization is limited as the department and the patient group may differ from other departments and patient groups in several regards. The department is highly specialized, has used the PRO-based follow-up approach for many years, and has referred a greater proportion of patients to PRO-based follow-up than other neurological departments in the region and in other regions of Denmark. Therefore, it is probable that the population of AmbuFlex/Epilepsy may be overrepresented by patients with severe epilepsy.

## Conclusion

Lower levels of health literacy (social support), well-being, self-efficacy and self-rated general health were associated with a greater need for contact to outpatient services at 1 year. Patients with lower levels of self-efficacy and fair general health perception also seem more likely to have inpatient contact and an emergency room visit. This study demonstrated that PRO measures may provided useful information in relation to the possibility of proactive efforts, prevention of disease-related issues and implementation of efficiency options regarding resource utilization. However, our findings would need to be confirmed in other epilepsy populations.

### Electronic supplementary material

Below is the link to the electronic supplementary material.

Supplementary Material 1: S1 Appendix

## Data Availability

Danish regulations prohibits us from making individual level data publicly available as the data contain potentially sensitive information. Researchers who are interested in replicating our work can apply for individual level data through the Office for Data Protection Central Denmark Region (Forskningsprojekter@rm.dk) – please refer to project reference number:1-16-02-691-14.

## Abbreviations

CI: Confidence Interval
GSE: The General Self-Efficacy Scale
HLQ: The Health Literacy Questionnaire
OR: Odds Ratio
PRO: Patient-Reported Outcome
SD: Standard Deviation
SF-36: The Short Form Health Survey
WHO-5: The WHO-Five Well-Being Index
