# Supplementary material for: Patient-reported outcome measures as determinants for the utilization of health care among outpatients with epilepsy: a prognostic cohort study
Source: J Patient Rep Outcomes. 2023 Oct 20;7:103. doi: 10.1186/s41687-023-00641-4 (PMC10589170; doi:10.1186/s41687-023-00641-4)
Supplement: Supplementary file 1 — Supplementary Material 1: S1 Appendix [file 41687_2023_641_MOESM1_ESM.docx]

| Table 5. Associations between the need for outpatient contact and PRO measures in outpatients with epilepsy without open access ^a^ | | | | |
| --- | --- | --- | --- | --- |
| Potential Determinants | **OR** | **95% CI** | **Adjusted OR ^b^** | **95% CI** |
| Social support for health (HLQ4 scores) | (n = 1,494)^c^ | | (n = 1,159)^c^ |  |
| High (score 3.8-4.0)  Medium (score 3.4-3.7)  Low (score 3.0-3.3)  Very low (score 1.0-2.9) | 1  1.02  1.06  1.81 | -  0.77 – 1.36  0.81 – 1.39  1.34 – 2.45 | 1  1.03  1.20  1.75 | -  0.74 – 1.45  0.87 – 1.65  1.21 – 2.53 |
| Ability to actively engage with health care providers (HLQ6 scores) | (n = 1,494)^c^ | | (n = 1,157)^c^ |  |
| High (score 4.4-5.0)  Medium (score 4.0-4.3)  Low (score 3.4-3.9)  Very low (score 1.0-3.3) | 1  1.10  1.76  1.86 | -  0.84 – 1.45  1.31 – 2.35  1.40 – 2.47 | 1  1.07  1.45  1.31 | -  0.76 – 1.49  1.03 – 2.05  0.92 – 1.86 |
| Understanding health information well enough to know what to do (HLQ9 scores) | (n = 1,492)^c^ | | (n = 1,163)^c^ |  |
| High (score 4.6-5.0)  Medium (score 4.0-4.5)  Low (score 3.4-3.9)  Very low (score 1.0-3.3) | 1  1.05  1.06  1.46 | -  0.80 – 1.37  0.78 – 1.43  1.07 – 1.97 | 1  1.17  0.81  1.08 | -  0.85 – 1.61  0.57 – 1.17  0.74 – 1.59 |
| Self-efficacy (GSE scores) | (n = 1,475)^c^ | | (n = 1,150)^c^ |  |
| High (score 33-40)  Medium (score 30-32)  Low (score 25-29)  Very low (score 10-24) | 1  1.13  1.57  1.72 | -  0.84 – 1.51  1.19 – 2.08  1.29 – 2.30 | 1  1.16  1.47  1.47 | -  0.83 – 1.63  1.06 – 2.05  1.02 – 2.10 |
| Well-being (WHO5 scores) | (n = 1,502)^c^ | | (n = 1,125)^c^ |  |
| High (score 70-100)  Medium (score 50-69)  Low (score 0-49) | 1  1.55  2.87 | -  1.22 – 1.98  2.12 – 3.87 | 1  1.48  2.23 | -  1.10 – 2.00  1.55 – 3.22 |
| General health (SF-36 first item categories) | (n = 1,596)^c^ | | (n = 1,187)^c^ |  |
| Excellent  Very good  Good  Fair  Poor | 1  0.88  1.29  2.79  3.88 | -  0.61 – 1.26  0.91 – 1.85  1.79 – 4.35  1.85 – 8.12 | 1  0.94  1.37  3.01  2.88 | -  0.60 – 1.47  0.87 – 2.16  1.71 – 5.30  1.16 – 7.14 |
| CI = Confidence Interval  ^a^ Total of 339 patients with open access  ^b^ Adjusted for age, gender, educational level, years with diagnose and seizure frequency  ^c^ Total numbers included in models as numbers vary due to missing values | | | | |

| Table 6. Associations between the need for inpatient contact and PRO measures in outpatients with epilepsy without open access ^a^ | | | | |
| --- | --- | --- | --- | --- |
| Potential Determinants | **OR** | **95% CI** | **Adjusted OR ^b^** | **95% CI** |
| Social support for helath (HLQ4 scores) | (n = 1,494)^c^ | | (n = 1,159)^c^ |  |
| High (score 3.8-4.0)  Medium (score 3.4-3.7)  Low (score 3.0-3.3)  Very low (score 1.0-2.9) | 1  1.39  0.95  1.32 | -  0.68 – 2.86  0.45 – 1.99  0.63 – 2.77 | 1  1.34  0.80  0.98 | -  0.59 – 3.06  0.33 – 1.94  0.40 – 2.40 |
| Ability to actively engage with health care providers (HLQ6 scores) | (n = 1,494)^c^ | | (n = 1,157)^c^ |  |
| High (score 4.4-5.0)  Medium (score 4.0-4.3)  Low (score 3.4-3.9)  Very low (score 1.0-3.3) | 1  0.72  1.24  1.13 | -  0.34 – 1.55  0.62 – 2.48  0.57 – 2.25 | 1  0.77  0.88  0.88 | -  0.32 – 1.87  0.37 – 2.12  0.37 – 2.10 |
| Understanding health information  (HLQ9 scores) | (n = 1,492)^c^ | | (n = 1,163)^c^ |  |
| High (score 4.6-5.0)  Medium (score 4.0-4.5)  Low (score 3.4-3.9)  Very low (score 1.0-3.3) | 1  1.15  0.90  1.15 | -  0.58 – 2.29  0.40 – 2.02  0.54 – 2.46 | 1  1.18  0.54  1.22 | -  0.53 – 2.63  0.19 – 1.51  0.48 – 3.11 |
| Self-efficacy (GSE scores) | (n = 1,475)^c^ | | (n = 1,150)^c^ |  |
| High (score 33-40)  Medium (score 30-32)  Low (score 25-29)  Very low (score 10-24) | 1  0.79  1.43  3.04 | -  0.30 – 2.07  0.65 – 3.16  1.48 – 6.21 | 1  0.64  1.19  2.29 | -  0.21 – 1.96  0.48 – 2.97  0.96 – 5.48 |
| Well-being (WHO5 scores) | (n = 1,502)^c^ | | (n = 1,125)^c^ |  |
| High (score 70-100)  Medium (score 50-69)  Low (score 0-49) | 1  1.22  1.85 | -  0.66 – 2.24  1.00 – 3.41 | 1  1.11  1.38 | -  0.52 – 2.39  0.63 – 3.04 |
| General health (SF-36 first item categories) | (n = 1,596)^c^ | | (n = 1,187)^c^ |  |
| Excellent  Very good  Good  Fair  Poor | 1  2.05  3.52  5.36  7.82 | -  0.46 – 9.05  0.83 – 14.91  1.20 – 23.95  1.47 – 41.64 | 1  3.27  4.10  7.36  6.17 | -  0.41 – 25.81  0.53 – 31.66  0.90 – 59.92  0.52 – 73.60 |
| CI = Confidence Interval  ^a^ Total of 339 patients with open access  ^b^ Adjusted for age, gender, educational level, years with diagnose and seizure frequency  ^c^ Total numbers included in models as numbers vary due to missing values | | | | |

| Table 7. Associations between the need for emergency room contact and PRO measures in outpatients with epilepsy without open access ^a^ | | | | |
| --- | --- | --- | --- | --- |
| Potential Determinants | **OR** | **95% CI** | **Adjusted OR ^b^** | **95% CI** |
| Social support for health (HLQ4 scores) | (n = 1,494)^c^ | | (n = 1,159)^c^ |  |
| High (score 3.8-4.0)  Medium (score 3.4-3.7)  Low (score 3.0-3.3)  Very low (score 1.0-2.9) | 1  1.51  1.13  1.55 | -  0.90 – 2.52  0.68 – 1.89  0.92 – 2.60 | 1  1.51  1.23  1.44 | -  0.84 – 2.74  0.68 – 2.24  0.78 – 2.66 |
| Ability to actively engage with health care providers (HLQ6 scores) | (n = 1,494)^c^ | | (n = 1,157)^c^ |  |
| High (score 4.4-5.0)  Medium (score 4.0-4.3)  Low (score 3.4-3.9)  Very low (score 1.0-3.3) | 1  0.68  0.90  1.23 | -  0.40 – 1.14  0.54 – 1.50  0.78 – 1.95 | 1  0.78  0.53  0.83 | -  0.44 – 1.36  0.28 – 1.00  0.47 – 1.48 |
| Understanding health information  (HLQ9 scores) | (n = 1,492)^c^ | | (n = 1,163)^c^ |  |
| High (score 4.6-5.0)  Medium (score 4.0-4.5)  Low (score 3.4-3.9)  Very low (score 1.0-3.3) | 1  0.97  0.74  1.37 | -  0.60 – 1.58  0.42 – 1.31  0.83 – 2.25 | 1  0.96  0.61  0.98 | -  0.56 – 1.65  0.32 – 1.17  0.53 – 1.82 |
| Self-efficacy (GSE scores) | (n = 1,475)^c^ | | (n = 1,150)^c^ |  |
| High (score 33-40)  Medium (score 30-32)  Low (score 25-29)  Very low (score 10-24) | 1  1.39  1.90  2.42 | -  0.77 – 2.50  1.11 – 3.23  1.43 – 4.10 | 1  1.05  1.76  1.76 | -  0.54 – 2.07  0.97 – 3.17  0.94 – 3.29 |
| Well-being (WHO5 scores) | (n = 1,502)^c^ | | (n = 1,125)^c^ |  |
| High (score 70-100)  Medium (score 50-69)  Low (score 0-49) | 1  1.09  1.63 | -  0.71 – 1.69  1.04 – 2.56 | 1  1.10  1.46 | -  0.65 – 1.86  0.85 – 2.51 |
| General health (SF-36 first item categories) | (n = 1,596)^c^ | | (n = 1,187)^c^ |  |
| Excellent  Very good  Good  Fair  Poor | 1  0.89  1.00  1.72  1.78 | -  0.45 – 1.74  0.52 – 1.92  0.84 – 3.52  0.66 – 4.78 | 1  1.18  1.19  2.45  1.81 | -  0.50 – 2.78  0.51 – 2.80  0.98 – 6.08  0.48 – 6.84 |
| CI = Confidence Interval  ^a^ Total of 339 patients with open access  ^b^ Adjusted for age, gender, educational level, years with diagnose and seizure frequency  ^c^ Total numbers included in models as numbers vary due to missing values | | | | |
